# Aortic dissection simulation models for clinical support: fluid-structure interaction vs. rigid wall models

**DOI:** 10.1186/s12938-015-0032-6

**Published:** 2015-04-15

**Authors:** Mona Alimohammadi, Joseph M Sherwood, Morad Karimpour, Obiekezie Agu, Stavroula Balabani, Vanessa Díaz-Zuccarini

**Affiliations:** Mechanical Engineering, University College London, Torrington Place, London, WC1E 7JE UK; Bioengineering, Imperial College London, South Kensington Campus, London, SW7 2BP UK; Mechanical Engineering, Imperial College London, South Kensington Campus, London, SW7 2AZ UK; Vascular Unit, University College Hospital, 235 Euston Road, London, NW1 2BU UK

**Keywords:** Fluid-structure interaction, Aortic dissection, Intimal flap, FSI, AD

## Abstract

**Background:**

The management and prognosis of aortic dissection (AD) is often challenging and the use of personalised computational models is being explored as a tool to improve clinical outcome. Including vessel wall motion in such simulations can provide more realistic and potentially accurate results, but requires significant additional computational resources, as well as expertise. With clinical translation as the final aim, trade-offs between complexity, speed and accuracy are inevitable. The present study explores whether modelling wall motion is worth the additional expense in the case of AD, by carrying out fluid-structure interaction (FSI) simulations based on a sample patient case.

**Methods:**

Patient-specific anatomical details were extracted from computed tomography images to provide the fluid domain, from which the vessel wall was extrapolated. Two-way fluid-structure interaction simulations were performed, with coupled Windkessel boundary conditions and hyperelastic wall properties. The blood was modelled using the Carreau-Yasuda viscosity model and turbulence was accounted for via a shear stress transport model. A simulation without wall motion (rigid wall) was carried out for comparison purposes.

**Results:**

The displacement of the vessel wall was comparable to reports from imaging studies in terms of intimal flap motion and contraction of the true lumen. Analysis of the haemodynamics around the proximal and distal false lumen in the FSI model showed complex flow structures caused by the expansion and contraction of the vessel wall. These flow patterns led to significantly different predictions of wall shear stress, particularly its oscillatory component, which were not captured by the rigid wall model.

**Conclusions:**

Through comparison with imaging data, the results of the present study indicate that the fluid-structure interaction methodology employed herein is appropriate for simulations of aortic dissection. Regions of high wall shear stress were not significantly altered by the wall motion, however, certain collocated regions of low and oscillatory wall shear stress which may be critical for disease progression were only identified in the FSI simulation. We conclude that, if patient-tailored simulations of aortic dissection are to be used as an interventional planning tool, then the additional complexity, expertise and computational expense required to model wall motion is indeed justified.

## Background

Aortic Dissection (AD) is a condition initiated by an intimal tear in the aortic wall. Haemodynamic forces cause the tear to progress, splitting the media layer and forming a ‘false-lumen’ (FL), wherein the blood flows between the layers of the vessel wall. Once an FL is formed, the disease has high mortality rates [1], and if untreated only 40% of patients suffering from type-B dissections (those involving only the descending aorta) will live longer than a month [2]. Such dissections are often treated medically (for example by pharmacologically lowering blood pressure with beta blockers [3]), but surgical interventions are necessary in the presence of complications such as malperfusion, downstream organ ischemia, severe hypertension, excessive enlargement of false lumen, leak and rupture [4,5]. However, surgery carries significant risk, including permanent spinal cord ischaemic damage, bleeding and mortality [4]. Endovascular interventions reduce the risk of complications, but their long term risk-factors are not yet clear [6].

The difficulties in selecting the most appropriate treatment for individual patients have led to an increased interest in patient-tailored computational approaches to aid the clinical decision making process; hence a great deal of research has been conducted with the common aim of constructing a framework which could be embedded within vascular clinics to provide clinicians with additional information that is not available from imaging data alone. Recent developments in medical imaging technology, such as 4D MRI, have enabled dynamic measurement of velocities and wall movement. However, further improvements in the technology are required in order to have sufficient resolution to accurately capture wall shear stress (WSS) and calculate pressure, particularly in the case of AD, where extremely low flow rates can be observed in the false lumen [7]. As an alternative, patient-tailored computational-fluid dynamics (CFD) approaches, wherein imaging data is used to construct a model of the patient’s aorta in which the flow is simulated, can provide data on WSS and pressure with high resolution [8-11]. Furthermore, the models once validated can be easily altered to evaluate the efficacy of various virtual intervention approaches [12-15]. However, as with most numerical simulations, these approaches require a number of assumptions to be made regarding the boundary conditions (pressure or flow, dynamic or static) and flow properties (viscosity model for blood, turbulence). Moreover, the aortic wall is compliant and the internal pressure gradients lead to the vessel geometry, and thus the flow domain, varying over the cardiac cycle. Fluid-structure interaction (FSI) simulations, which couple CFD simulations of the fluid with finite element modelling of the aortic wall, are capable of capturing this motion [16,17] but require specific expertise, are subject to further assumptions and critically, are prohibitively more computationally expensive. This warrants further investigation as to whether the extra effort required to incorporate the wall motion is justified in simulations of AD, in the context of clinical translation.

Taking these issues into account, this paper comprises a preliminary investigation into the application of FSI simulations in the computational analysis of a type-B aortic dissection.

## Methods

The present study is built on the patient-specific data analysed in our previous studies [11,12]. A 54 year old female patient with a history of hypertension was admitted to the hospital with chest and back pain. A CT scan confirmed a symptomatic type-B AD involving the origins of the arch branches, but not the ascending aorta. An initial attempt at medical management was made, however, with poorly controlled hypertension and worsening symptoms, the decision was made to proceed to surgery with the aim of covering the proximal entry tear.

The study was ethically approved (NHS Health Research Authority, ref: 13/EM/0143) and consent obtained. A CT scan of the aorta was performed prior to surgery using a 64 slice Siemens scanner (Siemens Healthcare, Erlangen, Germany). Invasive pressure measurements were made on the patient as part of the standard clinical procedure and the maxima and minima at each location were recorded (see [11] for details).

**Figure 1:**
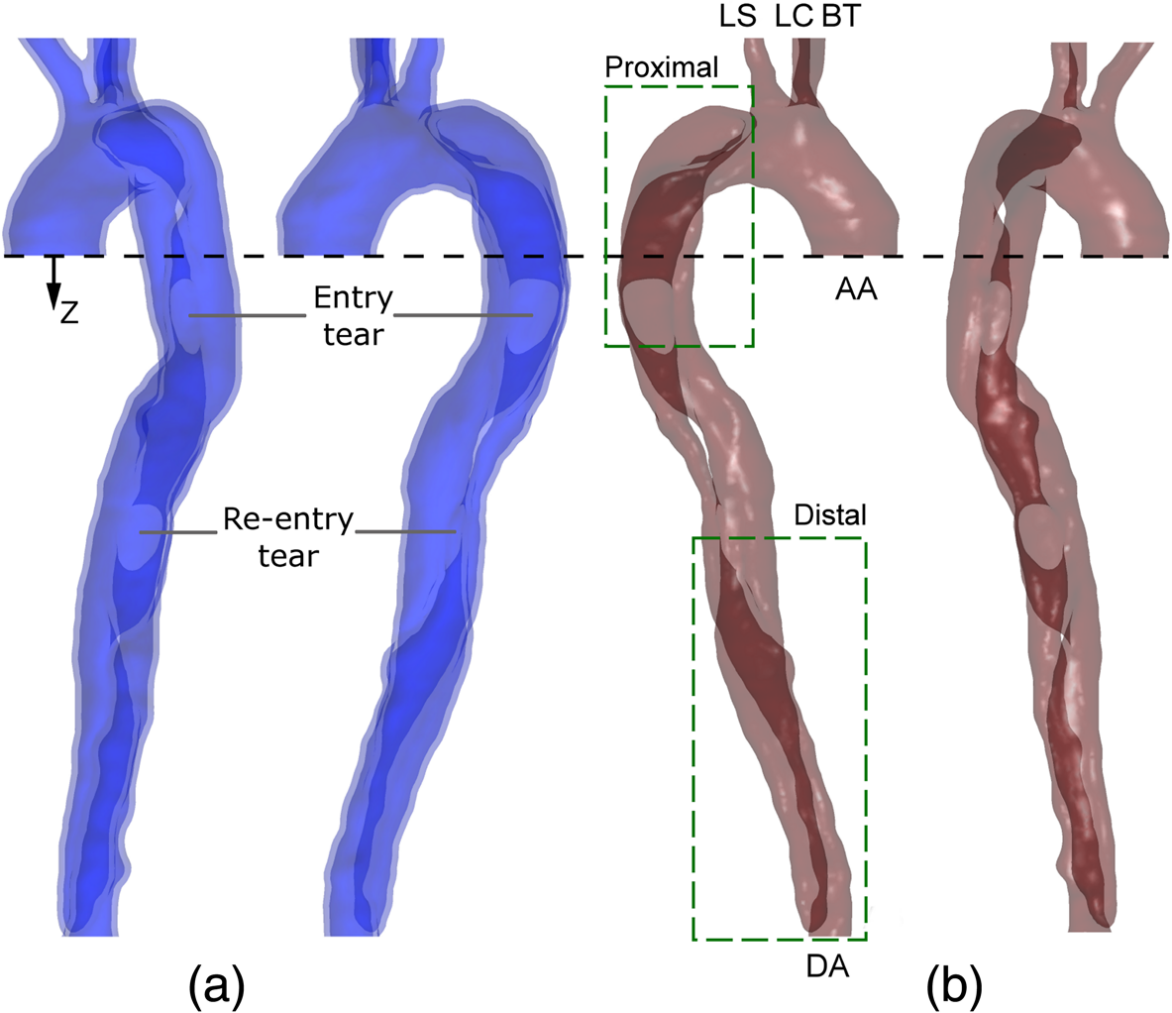
Geometry used for the present study. **(a)** Solid **(b)** Fluid. Entry and re-entry tears are indicated, as is the co-ordinate *z*. The domain boundaries are AA – ascending aorta; DA – distal abdominal; LS – left subclavian artery; LC – left common carotid; BT – brachiocephalic trunk. Dashed green boxes show the regions analysed in Figures 5 and 6.

The 3D fluid domain was generated from the CT images using ScanIP (Simpleware Ltd., Exeter, UK). A large aneurysm at the base of the subclavian artery was virtually removed, in order to provide a more general case for the analysis. The solid domain was created by extruding the outer wall of the fluid geometry to give an external thickness of 2.5 *mm*. The gap between the FL and TL was filled to create the intimal flap (IF) with a varying thickness along the dissected region of 2.45 ± 0.34 *mm* (median ± median absolute deviation). Figure 1a and b show the solid and fluid geometries respectively.

The blood was modelled as an incompressible fluid with a density of 1056 *kg/m*^3^ and non-Newtonian viscosity defined by the Carreau-Yasuda model with parameters from Gijsen et al. [18]. The shear-stress transport turbulence model (a hybrid k-ε, k-ω method) was applied [9,19,20], with 1% turbulence intensity at the inlet, similar to previous studies [9,21]. No flow data was available for the present patient, so data from a previous study was applied [22] at the inlet. Three-element Windkessel models were used at each of the fluid boundary outlets, with parameters calculated based on invasive pressure measurements from the same patient, as described previously [11]. A timestep of 5 *ms* was used; smaller timesteps did not significantly alter the results, but increased computational time.

The vessel wall was modelled using a hyperelastic model derived by Raghavan and Vorp [23]. An external pressure of 52.5 *mmHg* (the diastolic pressure in the dissected region) was applied. The centre point of each of the solid domain boundaries was fixed, and motion was restricted to the *xy*-plane only, as each of the boundaries is approximately normal to the flow direction, a similar approach to that of Brown et al. [16]. This allowed the boundaries to expand and contract with the pressure changes, but limited vertical translation of the model, which would be computationally expensive.

The fluid and solid domains were meshed using ANSYS ICEM-CFD (ANSYS Inc, Canonsburg, USA), and had approximately 230,000 and 50,000 elements, respectively. For the fluid mesh, 7 prismatic layers were included at the vessel wall. The simulation was run for three cardiac cycles, and a periodic state was reached after two cycles. The third cycle was extracted and used for the proceeding analysis.

For the purposes of comparison, an additional simulation was carried out with the same fluid mesh and boundary conditions, but without the solid domain, and with a fixed geometry. This will be referred to as the ‘rigid wall’ simulation herein.

Post-processing of the data was performed using ANSYS CFD-Post and MATLAB (Mathworks, Natick, USA). In order to analyse the variation of cross-sectional area of each lumen and how they are affected by the IF movement, an image processing methodology was utilised. At each timestep, a video of sequential *z*-planes along the descending aorta was generated. Each video was imported into LabVIEW (National Instruments, Austin, USA). An image processing methodology was implemented to identify the two lumina (TL and FL). For each *z*-plane at each timestep, the cross sectional area of each lumen was calculated and normalised by that in the rigid wall model, giving A^*^ = (*A*_*d*_-*A*_*u*_)/*A*_*u*_ *×* 100%, where the subscripts *d* and *u* represent deformed and undeformed (rigid) geometries respectively.

## Results

**Figure 2:**
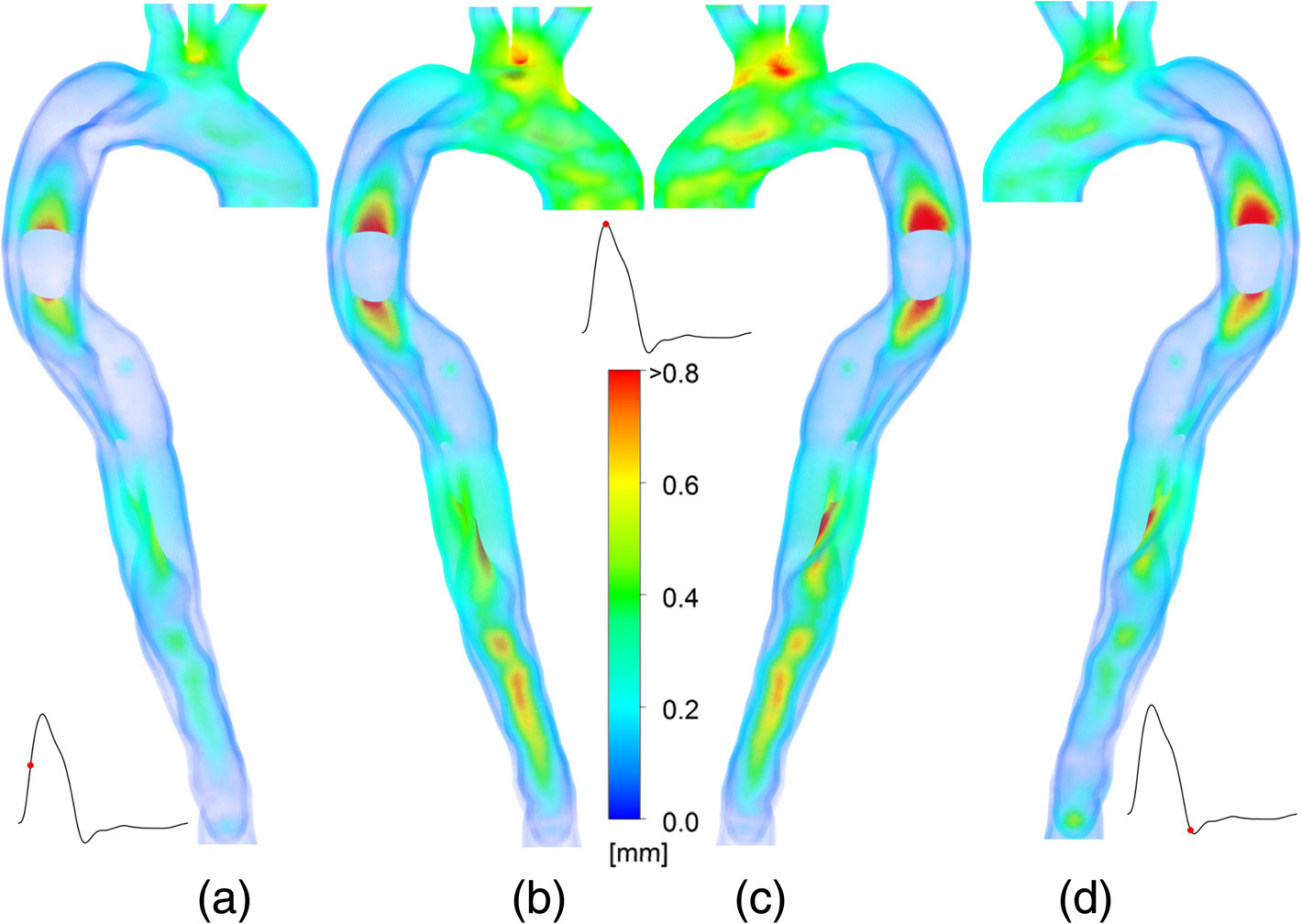
Displacement of the vessel wall at various time instances. Contours show the displacement relative to the undeformed geometry at **(a)** mid systole, **(b, c)** peak systole **(d)** dicrotic notch.

The displacement of the aortic wall is shown in Figure 2 for three time instances indicated in inset graphs. Figure 2a shows the displacement at mid systole. The ascending aorta, particularly around the branches has been displaced outwards, increasing the volume of the aorta. The edges of the entry tear are displaced by up to 0.75 *mm* and a displacement of the intimal flap (IF) distal to the re-entry tear of approximately 0.5 *mm* can be observed. At peak systole, two views are shown (Figure 2b and c). At this stage in the cardiac cycle, the ascending aorta has expanded further and the area dilation is ~ 7%. The intimal flap motion around the tear is further increased to >0.8 *mm*, and the IF distal to the re-entry tear is deformed considerably. At the dicrotic notch, (Figure 2d), the deformation around the entry tear is similar to that at peak systole, and distal IF has returned closer to its original position.

**Figure 3:**
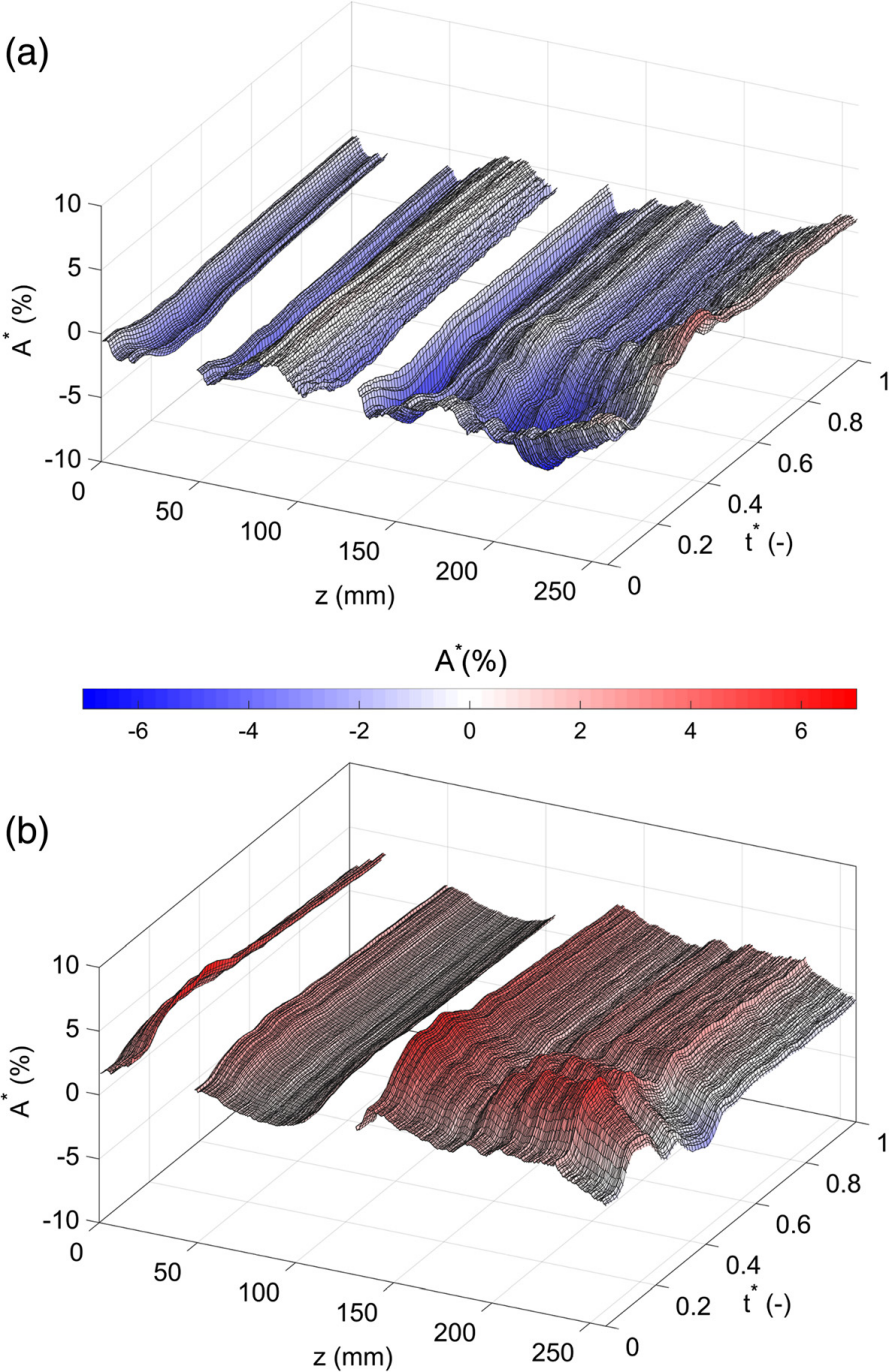
Map of lumina area ratio *A* ^*^. Maps of *A* ^*^ against *t* ^*^ (relative time in cardiac cycle) and *z* (see Figure 1 for reference). **(a)** True lumen, **(b)** False lumen.

In order to illustrate the deformation over time along the aorta, the lumina area ratio parameter A^*^ was utilised; this shows the instantaneous TL and FL cross-sectional areas along the descending aorta relative to the undeformed geometry. Figure 3a shows A^*^ as a function of *z* (defined relative to the inlet – see Figure 1) and t^*^ (the relative time instance in the cardiac cycle). Positive (red) values of A^*^ indicate an expansion of the cross sectional area, and negative (blue) values indicate a contraction. Gaps in the A^*^ maps correspond to the tear regions, wherein TL and FL are not defined. The true lumen cross-sectional area generally decreases by the motion of the IF. Proximal and distal to the entry tear, the deformation of the tear observed in Figure 2 can be seen throughout the cycle, with the greatest area contraction around systole. A region of small deformation exists between the two tears (*z* ~ 50-105 *mm*), but the IF deforms at the boundary of the re-entry tear, reducing the TL area. In the distal FL, there are large regions of considerable deformation, particularly around *z* = 200 *mm* at peak systole. At the very distal TL, the cross sectional area increases slightly at systole. As expected, the expansion of the FL is coupled with contraction of the TL, as shown in Figure 3b. The most striking change in cross sectional area takes place in the distal FL, which increases significantly in area over systole, and then relaxes rapidly. It should be noted that due to the tortuosity of the geometry, and the absence of a single luminal centerline from which to establish a normal direction, the A^*^ maps in Figure 3 are limited to the regions where the axes of the TL and FL are approximately parallel to the *z*-axis, and thus do not cover the deformation of the proximal FL.

From the above observations, it becomes clear that the absence of flap motion in the rigid wall simulations is likely to influence the estimated haemodynamic parameters, which highlights the value of FSI simulations in AD. However, in order to address the question of whether FSI simulations are worth the effort, the significance of the differences in estimated parameters from the two types of simulations needs to be established in relation to the clinically useful information required.

**Figure 4:**
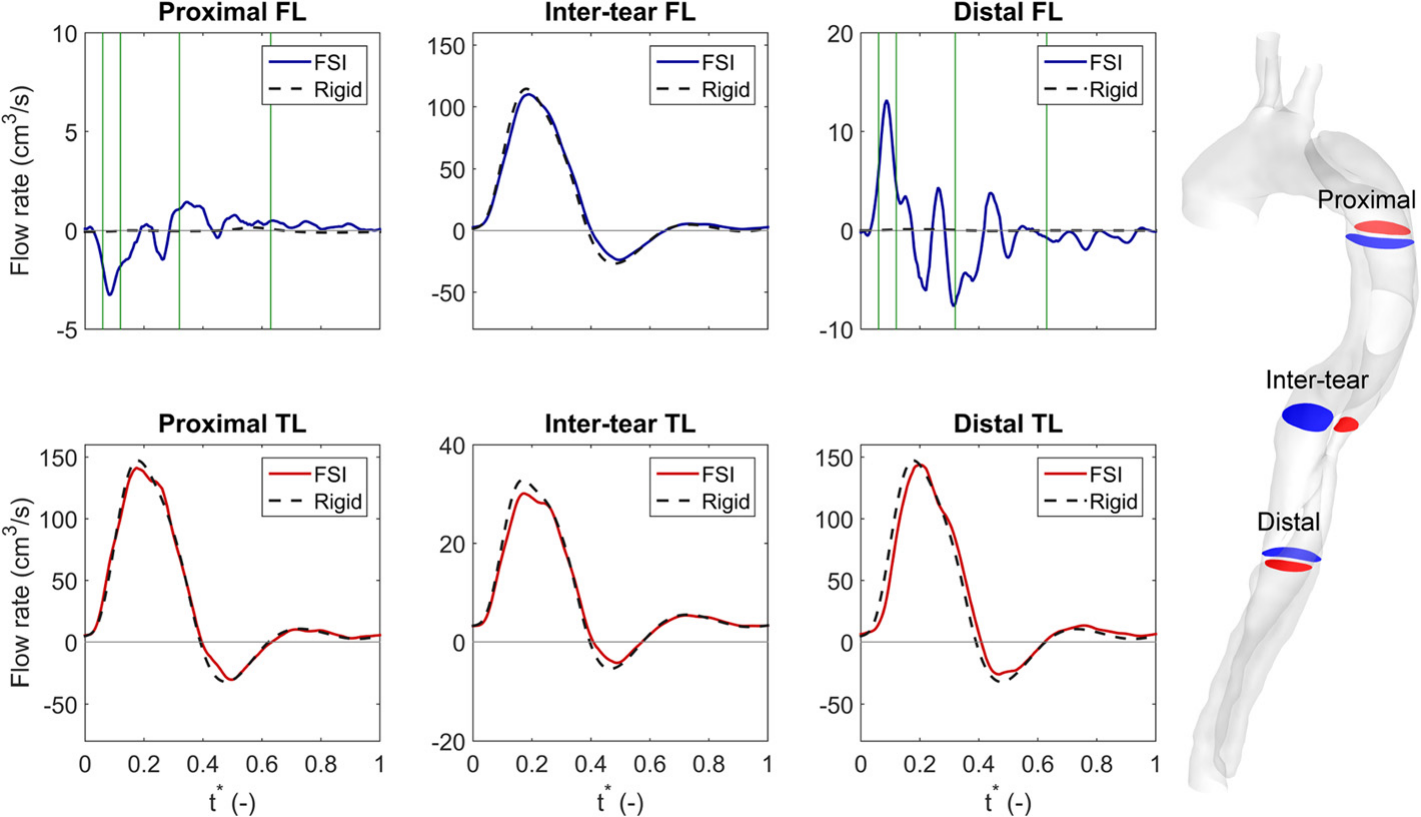
Flow distributions in the descending aorta. Each subfigure compares the flow rate across the indicated plane for FSI and rigid wall simulations against *t* ^*^. Red - true lumen, blue - false lumen. Vertical green lines in Proximal FL and Distal FL subfigures indicate time instances considered in Figures 5 and 6.

Figure 4 investigates the effects of the IF motion on the proportion of flow going through each lumina at three planes: proximal to the first tear (‘Proximal’), between the two tears (‘Inter-tear’) and distal to the re-entry tear (‘Distal’). For each plane, the flow rate through the two lumina obtained from the FSI simulation is shown by solid lines, with the rigid wall simulation results shown by the dotted black lines. In the proximal TL, it can be seen that the shape of the flow waves is similar for the FSI and rigid wall simulations, although the compliance of the vessel wall in the former induces a small phase lag in the flow. However, these differences are not expected to be significant in a clinical context. Similar observations can be made regarding the flow in the inter-tear and distal TL. In between the two tears (inter-tear), the majority of the flow is actually going through the FL. This is unsurprising given the relatively larger cross sectional area in this region, but is larger than the 40% of FL flow reported by Chen et al. [19].

The flow rates obtained from FSI simulations in the proximal and distal FL, are considerably different to those obtained with the rigid wall model; a small (note the different *y-axes*), but not insignificant amount of flow crosses the selected planes in the false lumen, whereas the rigid wall model predicts almost completely stagnant flow. In the proximal FL, the flow is at minimum at mid systole, then oscillates between positive and negative flow until about halfway through the cardiac cycle, after which little net flow exists. Note that due to the co-ordinate system defined in Figure 1a, negative flow corresponds to fluid entering the proximal FL.

The flow in the distal FL is of higher magnitude than the proximal FL, and appears to be out of phase with the proximal FL, i.e. as the flow in the proximal FL decreases, the distal FL increases and vice versa. However, due to the co-ordinate system, Figure 4 actually shows that both of these regions expand and contract in phase.

**Figure 5:**
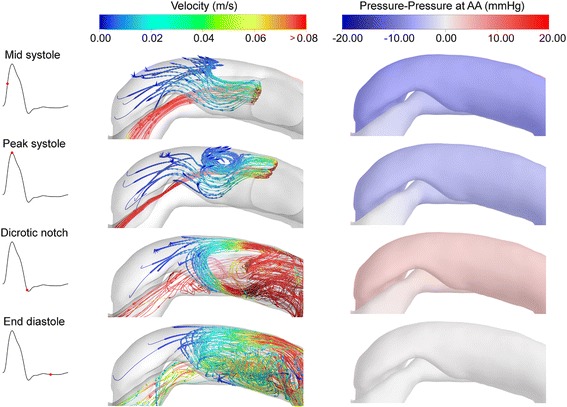
Flow characteristics in the proximal FL. Left column shows streamlines calculated from the ‘Proximal’ plane in the FL (see Figure 4). Each row shows a time instance as indicated by the insets and the vertical green lines in Figure 4. Right hand column shows pressure contours relative to the pressure at the AA.

Given that the total net flow over the cardiac cycle in the upstream and downstream FL must be zero, there is negligible flow in the rigid wall simulation. However, when the wall motion is considered, the area of these regions will vary throughout the cardiac cycle, which results in fluid being drawn in and expelled accordingly, depending on the pressures and wall motions. Figure 5 investigates these dynamics for the proximal false lumen, over the region indicated in Figure 1b. In addition to the three time instances shown in Figure 2, a fourth time instance at end diastole is shown, as indicated by the vertical green lines in Figure 4a. The right column shows pressure relative to that at the AA (the fluid inlet), so as to illustrate the pressure gradient. The left hand column shows streamlines, which are calculated in both directions (forward and backward) from seed points located on the Upstream FL plane (blue) shown in Figure 4, thereby showing the possible source/destination of the flow in the region of interest. At mid systole, a small amount of fluid passing through the coarctation (narrowed region) moves into the proximal FL, although the flow in the most proximal part is small. At this point, there is a slight pressure gradient between the TL and FL, which may be the driving force for this flow. At peak systole, the pressure gradient is similar and a vortex has developed close to the tear. Interestingly, the separation surface dividing fluid passing into the proximal FL from the mid TL and FL, has offset to the side of the tear. As in mid systole, there is a small amount of flow into the most proximal part of the FL. At the dicrotic notch, the pressure gradient is inverted, but is of small magnitude (about 5 *mmHg*). The flow around the tear becomes chaotic and the vortex increases in size, extending into the tear region. The small amount of fluid that entered the proximal FL during systole is drawn into the vortex and thus out of the proximal FL. At end diastole, there is negligible fluid movement in the proximal FL, and the flow in the tear region is very chaotic. The vortical flow structure around the tear in the proximal FL may explain the oscillations in net flow observed in Figure 4.

**Figure 6:**
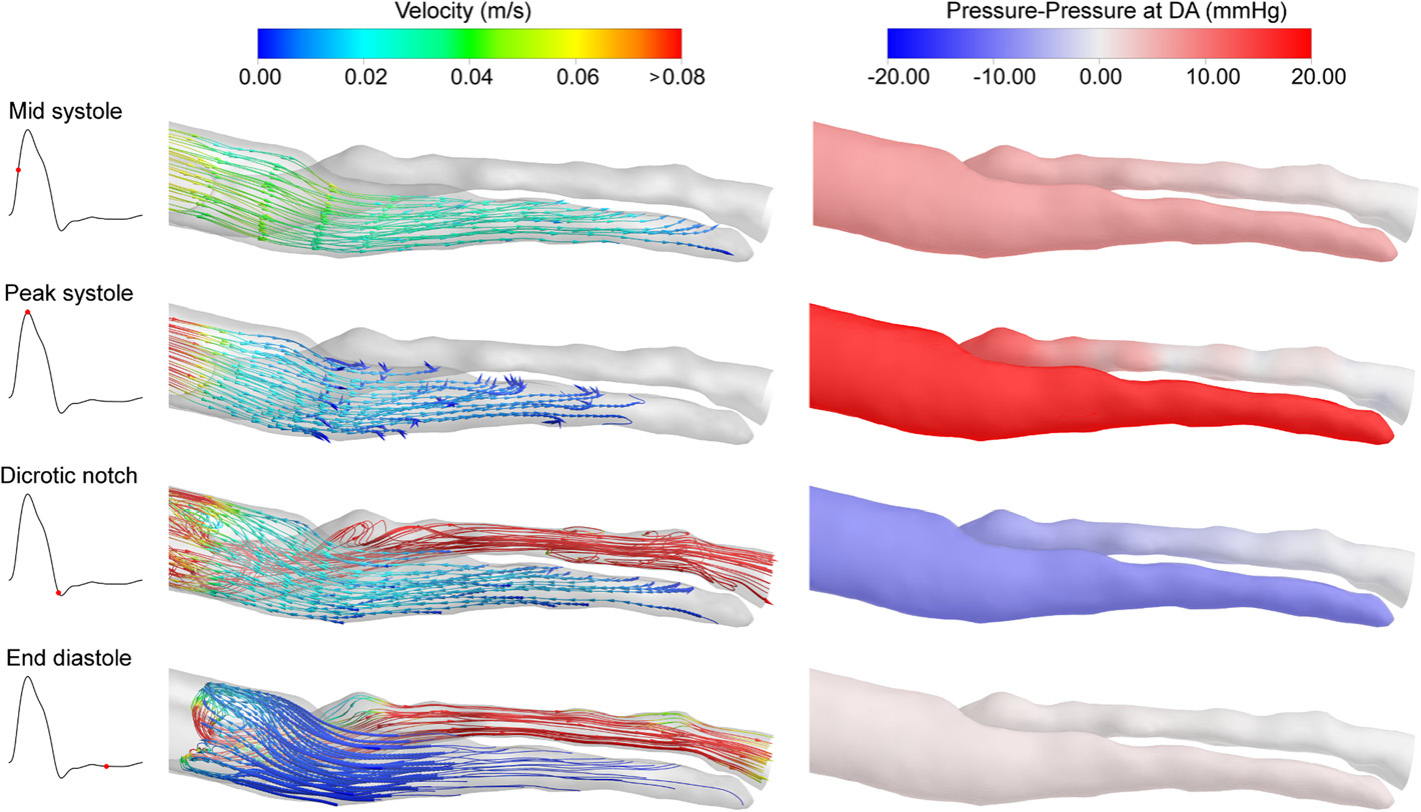
Flow characteristics in the distal FL. Left column shows streamlines calculated from the ‘Distal’ plane in the FL (see Figure 4). Each row shows a time instance as indicated by the insets and the vertical green lines in Figure 4. Right hand column shows pressure contours relative to the pressure at the DA.

Figure 6 shows the corresponding distributions for the distal FL (see Figure 1b). The dynamics in this region are significantly different to the proximal FL. At mid systole, flow passing through the Middle FL continues directly into the distal FL, and the streamlines extend to the very bottom of the region. At peak systole, there is a large pressure gradient of almost 20 *mmHg* between the FL and TL, which could be expected to generate considerable flow between the two. Conversely, the flow still enters the distal FL at this time instant, albeit at a relatively low flow rate. At the dicrotic notch, the pressure gradient is inverted, but the fluid in the distal FL is ejected as the pressure decreases and the wall contracts. At end diastole, the velocity in the distal FL is almost zero and the pressures between the lumina are approximately equal.

**Figure 7:**
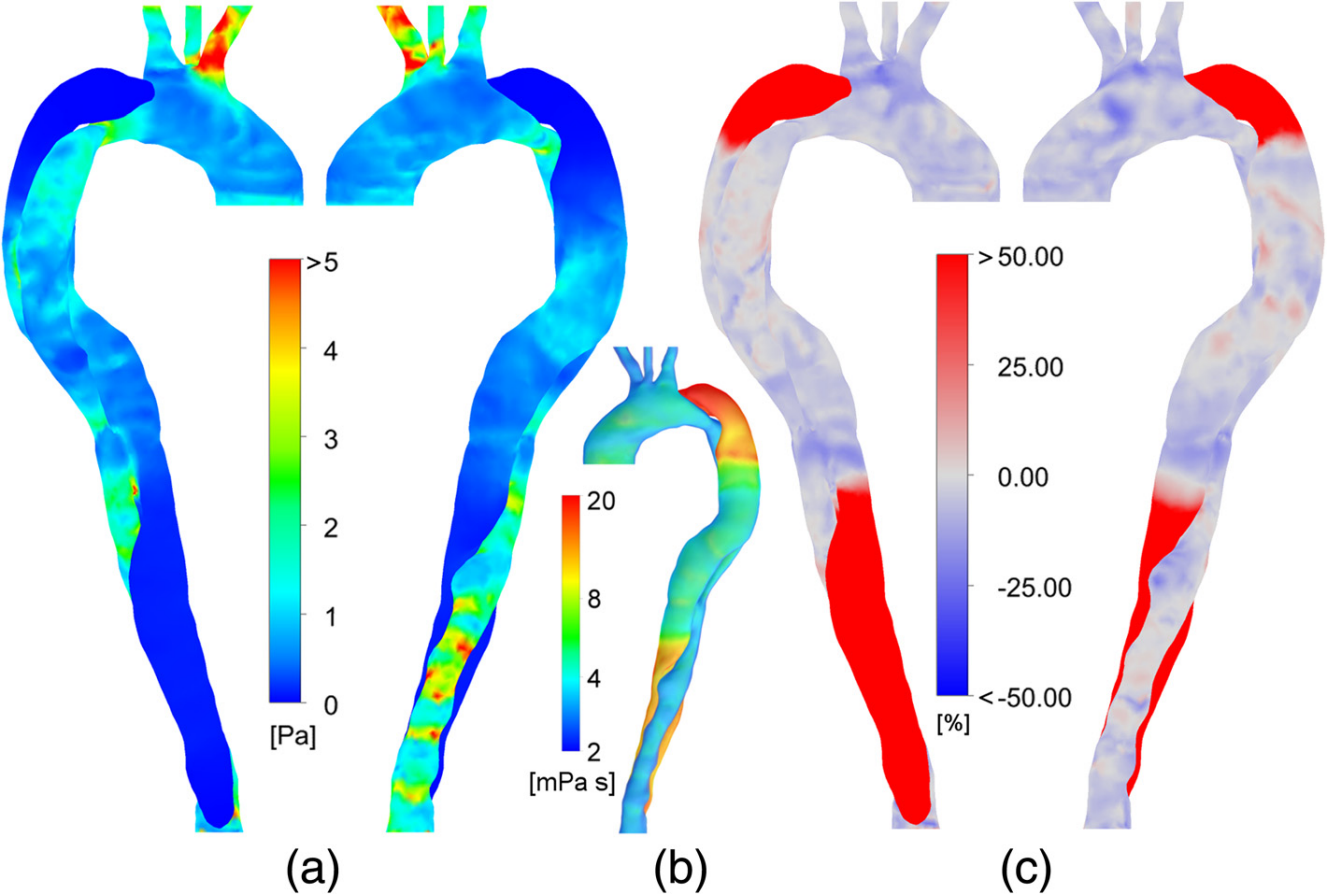
Wall shear stress characteristics. **(a)** TAWSS distributions for the FSI simulation, **(b)** Dynamic viscosity distribution at peak systole, **(c)** Percentage difference in TAWSS relative to the rigid wall model.

It is apparent that these complex dynamics cannot be captured with rigid wall simulations, but the question remains as to whether the additional simulation effort expended in gaining this resolution is necessary. One of the key outputs from these simulations is prediction of the wall shear stress, which is typically analysed using various indices, such as the time-averaged wall shear stress (TAWSS) and oscillatory shear index (OSI) [24]. The time-averaged wall shear stress shows the average magnitude of the wall shear stress over the cardiac cycle, and its distribution obtained with FSI simulations is shown in Figure 7a.

High values of TAWSS can be observed in the aortic branches and along the distal TL. In the three branches, there is more flow than would normally be expected in a healthy aorta, due to the increased hydrodynamic resistance of the pathological thoracic aorta. This results in elevated values of TAWSS in the branches. Despite the reduced flow rate through the distal TL (see [11] for further discussion), the local velocities are increased due to its geometric constriction, and hence elevated TAWSS values are observed. In the proximal and distal FL regions, the TAWSS is almost zero. The high values of viscosity in this region (Figure 7b), as predicted by the Carreau-Yasuda model for the shear-thinning properties of blood, may be responsible for reducing velocities in this region, which would decrease WSS. However, the higher viscosity would act to increase WSS (as WSS is defined as velocity gradient multiplied by local viscosity).

The percent change in the calculated TAWSS as a result of considering the wall motion in the simulation is shown in Figure 7c. Throughout the true lumen, a difference of around -20% can be observed, indicating that the rigid wall model slightly overestimates the TAWSS. In the distal and proximal FL however, a large difference of significantly more than 50% can be seen (colour axes are limited to ± 50% for clarity). This is due to the near zero velocity values observed in the rigid wall simulation, which appears to have a notable impact on the TAWSS values. However, in absolute terms (approximately ± 0.2 *Pa*), it is clear that the differences between the rigid wall and FSI models are not that significant and if only TAWSS was of interest, FSI modelling might not be required.

**Figure 8:**
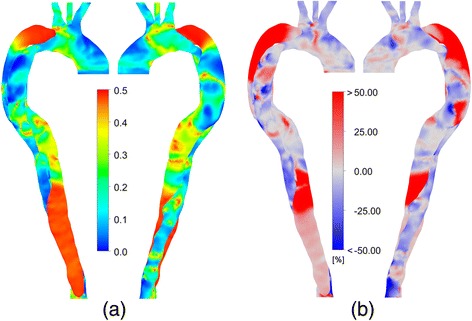
OSI characteristics. **(a)** OSI distributions for the FSI simulation, **(b)** Percentage difference in OSI relative to the rigid wall model.

Figure 8a considers the oscillatory shear index, a measure of the oscillatory nature of the flow. This index varies considerably throughout the domain, except in the distal and proximal false lumen, where it exhibits consistently high values, meaning that the directional changes are large relative to the mean flow. When considered along with Figure 7, it can be observed that these regions appear to exhibit both high OSI and low wall shear stress, which has been identified as a potential index of high risk regions for aneurysm rupture [25,26]. Figure 8b shows the percentage change in the calculated OSI due to wall motion. It can be seen that there are considerable differences in the proximal and distal FL (approximately ± 0.15 in absolute terms), where the complex dynamics analysed in Figures 5 and 6 significantly enhance the oscillatory nature of the flow.

## Discussion

The present study investigated whether the additional complexity, and critically additional time, required to perform patient-tailored FSI simulations when modelling AD as a tool for interventional planning is justified. The rigid wall simulation took just 12 hours to complete 3 cycles and reach the periodic state, in contrast to the 170 hours required for the FSI simulations on a desktop computer (Intel i7 processor, 32 GB RAM). Although using a more powerful computer would decrease the simulation time, standard computing tools will still struggle to complete the FSI simulation in less than a few days, which may be too long in the case of AD, in which timely intervention is often critical.

There is a growing amount of literature on FSI simulations of aortae [16,17,27-29], using various models for the structure of the vessel wall [30,31] in order to capture the movement as effectively as possible. However, there is no consensus on how best to define vessel wall properties from imaging data alone as yet. This difficulty is exacerbated in the case of AD, which is often accompanied by increased vessel wall stiffness [32], which may not be evenly distributed throughout the domain. The present simulation uses a simple hyperelastic model extracted from experimental data on an aneurysm, and a uniform wall thickness throughout (except the intimal flap) and as such may not perfectly capture the wall dynamics. However, the results showed deformations quantitatively similar to those measured *in vivo* by other researchers. The change in cross-sectional area of the TL and FL was calculated from ECG-gated CT scans by Ganten et al. [33] and they observed a mean TL cross-sectional area reduction (-4.4%), comparable to the A^*^ values shown in Figure 3a. The median IF oscillation observed by Ganten et al. [33] was 1.3 *mm*, while Karmonik et al. [34] reported average IF displacements of approximately 0.48-0.68 *mm* from MRI data. These values compare favourably with the IF deformation shown in Figure 2. In a recent paper, Yang et al. [35] observed much larger flap motions (1.8 *mm* – 10.2 *mm*). It is not clear at present why such a discrepancy exists between these data and the aforementioned imaging studies. In an FSI simulation of a simplified dissected aorta, Qiao et al. [15], found flap displacements of only up to 0.15 *mm*, which may be attributable to the use of a Young’s modulus of 100 *MPa*.

This study provided high-resolution data, from which a detailed ‘map’ of cross-sectional area changes was generated. Such a map provides an intuitive and simple way to consider the geometric locations where the vessel wall motion is most significant, and thus has the potential to be a useful clinical tool. 4D MRI data would provide a more rigorous validation of the estimated wall motion. However, such time resolved data was not available for the patient geometry studied here and are not routinely acquired in the clinic; hence the similarities between the simulated values and those reported elsewhere provides confidence that the model employed in this preliminary study is appropriate.

The pathophysiology of aortic dissection is not well understood, and thus it is not entirely clear what the most useful data to extract from such FSI simulations is. Longitudinal studies involving a large number of patients are required to identify critical parameters/disease markers and establish benchmarks for clinical use. However, in the absence of such direct information, and given that dissections form from aneurysms [3], it is reasonable to assume that the pathophysiology of AD may be similar to aneurysms.

Evidence suggests that regions of high wall shear stress (WSS) are implicated in AD; for example, it has been reported that a reduction in shear stress can minimise the propagation of the dissection [2]. Additionally, it has been found that initial tear locations are coincident with regions of maximal pressure or WSS [36]. Thubrikar et al. [37] also correlated elevated WSS with sites of intimal tears. The locations of the regions of high TAWSS observed in Figure 7 were not significantly altered in the FSI simulation, compared to the rigid wall.

However, a considerable amount of evidence also suggests that in the context of aneurysms, regions of collocated high OSI and low TAWSS are of particular risk of rupture [25], calcification [38] or wall thickening [36]. As reviewed by Meng et al. [26], in such regions, multifaceted endothelial dysfunction is observed, including increased permeability and ‘stickiness’ along with inflammatory responses [25,26,38,39].

The proximal and distal FL in the present simulation exhibit both of these characteristics, indicating that they are the regions of most significant risk. It was established in this work that the rigid wall model was not capable of accurately capturing the fluid motion in these regions. Furthermore, 4D MRI methods are not yet capable of accurately resolving the slow but complex flows in the FL [7]. Hence, it can be concluded that numerical tools are a promising option for analysing haemodynamics in AD, so long as wall motion is included in the simulation.

If it transpires that the imaging data of Yang et al. [35] is more characteristic of true flap motion than previous reports [33,34], then it is clear that the loss of accuracy through the rigid wall simplification would be exacerbated. However, further developments are required, particularly in calculating the vessel wall properties of a dissected aorta, before this technology will be able to truly predict the fluid and solid dynamics of this condition. Once such models have been developed and validated, it will be possible to accurately estimate the internal stresses in the wall of a dissected aorta, which may provide further clinical indicators [40].

In addition, the complex behaviour of blood viscosity is likely to play a greater role in AD than in other aortic diseases, as highlighted in Figure 7b. There is no consensus as to what constitutes the best non-Newtonian viscosity model for blood, perhaps due to the absence of high-resolution experimental data of blood in appropriate geometries. However, the Carreau-Yasuda model with parameters according to Gijsen et al. [18] has been widely cited, and was thus used in this study. The influence of the non-Newtonian viscosity characteristics can be observed in Figure 7b, with reduced viscosity at the vessel walls in the TL (due to high local shear rates) and high viscosity in the proximal and distal FL, due to the very low shear rates. Therefore, we conclude that, in addition to FSI, non-Newtonian fluid models should be used in AD simulations.

Although this study uses some patient-specific data; the geometry obtained from patient CT scans and the invasive pressure measurements (minima and maxima) that were used to tune the Windkessel outlets), it does not aim to be a fully patient-specific simulation, but rather a sample representation of the condition. Turbulence, non-Newtonian blood viscosity and non-linear wall mechanics were accounted for in the simulation, with the aim of representing the biomechanical environment as accurately as possible. Each of these additions made to the simulation as compared to Newtonian, laminar flow with linear elastic walls introduces a degree of dependence of the results on the parameters selected and hence uncertainty. The comparable levels of wall displacement provide some validation of the model, but are not sufficient to allow for rigorous parameter estimation. The influence of the selected parameters is discussed in the following paragraphs.

Although no flow data from the patient was available for the inlet flow profile, necessitating the use of data from another patient, Windkessel parameters tuned to invasive pressure measurements from the patient were used, resulting in a pressure environment (of particular importance when modelling wall motion) that was relatively representative of the patient. As only the pressure minima and maxima were available, the Windkessel parameter combinations used in this study are not unique, but were shown to be fairly robust to small changes in the pressure measurements [11], and the use of dynamic boundary conditions is a significant improvement of constant pressure or flow-split alternatives used in previous studies. The fluid modelling used in this study incorporated both turbulence modelling and non-Newtonian viscosity models. Although it has still not been established how best to model blood in the aorta, studies have shown that including turbulence allows slightly improved estimates of blood velocity when compared with pcMRI data [20,21]. Cheng et al. [9] used the SST Tran model, which has been developed to model transitional flows, which may be more appropriate than a fully turbulent model for aortic blood flow, but this requires two additional equations to be solved and thus the SST model was used in the present study for computational efficiency. It is well established that blood is non-Newtonian, and in the low flow regions of the proximal and distal FL, this is expected to be of particular importance. For this work, the use of the Carreau-Yasuda model can thus be expected to improve the model.

The model of Raghavan and Vorp [23] was selected to simulate the wall mechanics over a linear elastic model. This model was developed for abdominal aortic aneurysms, which could be expected to be comparable to aortic dissection. Although the solution will be partly dependent on the choice of material properties for the wall, a large range of Young’s moduli have been reported in the literature, from 0.4 to 6 *MPa* [28,41,42] and it is not clear what material properties are most appropriate for modelling the arterial wall, particularly in AD. In the small strain range (<0.03) observed in the present study, the model of Raghavan and Vorp [23] is not significantly different from a linear elastic model with a Young’s modulus of 1 *MPa*, as used for example by Brown et al. [16], but provides additional numerical robustness in this case.

Finally, the mesh sensitivity should be mentioned. It was not possible to generate a mesh of sufficiently high quality for the complex wall geometry using fewer than 50,000 elements, and so only the influence of refinement of both the solid and fluid meshes was analysed. A fine mesh was generated that contained approximately 150,000 solid elements and 530,000 fluid elements. Comparisons between the fine mesh and the one used in this study (medium mesh) showed that the predicted wall displacements were slightly reduced with the finer mesh, but by less than 0.1 *mm* throughout the majority of the domain and by less than 0.2 *mm* in the ascending arch. A direct comparison of the WSS indices, TAWSS and OSI, showed only minor differences between the two meshes that are unlikely to affect the clinical interpretation of the results (not shown for brevity). The velocities obtained with the two meshes were also similar: at peak systole, the differences throughout the domain were less than 0.05 *m/s,* except in the supraaortic branches wherein the medium mesh under-predicted the velocity by 0.15 *m/s*, as compared to the approximately 2.5 *m/s* in these branches at this time point. Furthermore, the fine mesh took 2.7 times longer to run than the medium mesh. It can be concluded this additional computational expense did not yield significantly improved results, and thus the ‘medium’ mesh density used is appropriate.

## Conclusions

The present study comprises the most advanced model of haemodynamics in a dissected aorta reported in the literature to date, with coupled dynamic boundary conditions, non-Newtonian viscosity, turbulence and wall motion all accounted for. From the comparison with rigid wall simulations, we conclude that in order to develop meaningful information for clinicians dealing with AD, wall motion is a necessary component of the model, without which key regions of interest may not be accurately captured. Despite the additional computational cost, the present study shows that this is an important factor in the model, as well as accurate modelling of the blood viscosity and application of appropriate boundary conditions. Further work is required to establish the mechanical properties of dissected aortae in order to take the current model to the next level of accuracy; however, this is also likely to further increase the computational time. It may transpire that a reasonable compromise for the clinic would be to use 4D imaging data to impose the vessel wall motion, and analyse the haemodynamics. Comparing such results with full FSI studies in a research setting could establish the viability of this approach.

## Abbreviations

FSI: Fluid-structure interaction
CFD: Computational fluid dynamics
AD: Aortic dissection
AA: Ascending aorta
DA: Distal abdominal
BT: Brachiocephalic trunk
LC: Left common carotid artery
LS: Left subclavian artery
